# Layer Specific Development of Neocortical Pyramidal to Fast Spiking Cell Synapses

**DOI:** 10.3389/fncel.2015.00518

**Published:** 2016-01-20

**Authors:** Olga Voinova, Fliza Valiullina, Yulia Zakharova, Marat Mukhtarov, Andreas Draguhn, Andrei Rozov

**Affiliations:** ^1^Department of Clinical Neurobiology, University of HeidelbergHeidelberg, Germany; ^2^OpenLab of Neurobiology, Kazan Federal UniversityKazan, Russia; ^3^Department of Physiology and Pathophysiology, University of HeidelbergHeidelberg, Germany

**Keywords:** development, neocortex, synapses, excitation, interneurons

## Abstract

All cortical neurons are engaged in inhibitory feedback loops which ensure excitation-inhibition balance and are key elements for the development of coherent network activity. The resulting network patterns are strongly dependent on the strength and dynamic properties of these excitatory-inhibitory loops which show pronounced regional and developmental diversity. Therefore we compared the properties and postnatal maturation of two different synapses between rat neocortical pyramidal cells (layer 2/3 and layer 5, respectively) and fast spiking (FS) interneurons in the corresponding layer. At P14, both synapses showed synaptic depression upon repetitive activation. Synaptic release properties between layer 2/3 pyramidal cells and FS cells were stable from P14 to P28. In contrast, layer 5 pyramidal to FS cell connections showed a significant increase in paired pulse ratio by P28. Presynaptic calcium dynamics also changed at these synapses, including sensitivity to exogenously loaded calcium buffers and expression of presynaptic calcium channel subtypes. These results underline the large variety of properties at different, yet similar, synapses in the neocortex. They also suggest that postnatal maturation of the brain goes along with increasing differences between synaptically driven network activity in layer 5 and layer 2/3.

## Introduction

Many synapses change their efficacy upon repetitive activation. This short-term plasticity is an important determinant of neuronal signal integration and spatiotemporal pattern formation in neuronal networks (Zucker and Regehr, 2002). The underlying mechanisms are mostly related to vesicular release probability and calcium dynamics in presynaptic terminals. Several synapses have been shown to undergo developmental changes in short-term plasticity which, in turn, may contribute to the maturation of coherent network behavior. For example, paired pulse facilitation (PPF) of excitatory postsynaptic potentials (EPSPs) at Schaffer collateral inputs to CA1 pyramidal neurons increases with age, while transmitter release probability decreases (Muller et al., 1989; Bolshakov and Siegelbaum, 1995). At reciprocal connections between neocortical layer 2/3 pyramidal cells there is a switch in the mode of short term plasticity, from paired pulse depression (PPD) in P14 rats to PPF by the end of the 4th postnatal week (Reyes and Sakmann, 1999). Similar changes in dynamic properties have also been observed at synapses between layer 5 thick tufted pyramidal neurons (Reyes and Sakmann, 1999) and at slender tufted pyramidal cell connections (Frick et al., 2007). Developmental modulation of synaptic release properties, and consequently short-term plasticity modes, is not confined to excitatory synapses between pyramidal neurons. During the 3rd and 4th postnatal weeks, a variety of excitatory inputs onto GABAergic neurons have also been shown to undergo changes in release properties. In striatal medium spiny neurons there is a substantial increase in PPF of EPSPs by P20 (Choi and Lovinger, 1997). Angulo et al. (1999) clearly showed that connections between layer 5 pyramidal cells and fast spiking (FS) interneurons exhibit PPD over a wide range of stimulation frequencies in young animals. However, the same group also demonstrated that starting from the 5th postnatal week, the majority of pyramidal to FS neuron connections switch to PPF at low stimulation rates (0.2 Hz), while PPD persists at higher rates (1 Hz). They suggest that this developmental switch from PPD to PPF at low stimulation rates (0.2 Hz) arises due to modifications in the presynaptic terminal.

FS basket interneurons play a key role in the generation of cortical gamma oscillations (Buzsaki and Wang, 2012). In rodents, gamma oscillations emerge during the second postnatal week (Lahtinen et al., 2002; Leinekugel et al., 2002; Doischer et al., 2008).

However, gamma patterns characteristic for adult rats are fully developed only by the end of the first postnatal month (Colonnese et al., 2010; Minlebaev et al., 2011; Khazipov et al., 2013). This delayed development of gamma oscillations is likely to reflect the delayed maturation of the feedback loop formed by local pyramidal cells and FS interneurons (Du et al., 1996; Angulo et al., 1999; Chattopadhyaya et al., 2004; Okaty et al., 2009; Wang and Gao, 2010; Yang et al., 2014).

Therefore, the three major aims in this study were: (i) to find out whether synaptic efficacy and synaptic plasticity at local layer 2/3 pyramidal to FS cell synapses can be developmentally regulated similarly to that in layer 5; (ii) to compare release properties in the pyramidal to FS cell terminals located in different cortical layers; and (iii) to find out the structural differences underlying the change in release properties at layer 5 pyramidal to FS cell synapses. Taking into account the importance of the second two postnatal weeks (P14–P28) for the maturation of neocortical gamma oscillations, in this study we analyzed the properties of local excitatory inputs to FS interneurons at these two ages. We compared vesicular release properties at intralaminar synaptic connections between either layer 2/3 or layer 5 pyramidal cells and FS interneurons during the critical developmental period between the second and fourth postnatal week (P14 and P28, respectively). We investigated whether synaptic transmission from pyramidal cells to FS interneurons differs between different layers and developmental stages of the rat neocortex. Finally, we identified possible mechanisms underlying the observed differences. Our findings point towards an increasing difference in synaptic feedback loops and frequency-dependent synaptic communication within the two cortical layers. Such differences are likely to contribute to the maturation of complex inter- and intralaminar patterns of network activity (Whittington et al., 2011).

## Materials and Methods

All experimental protocols were performed in accordance with Kazan Federal University on the use of laboratory animals (ethical approval by the Institutional Animal Care and Use Committee of Kazan State Medical University, N9–2013) or by the state government of Baden-Württemberg, Germany. All efforts were made to minimize animal suffering and to reduce the number of animals used.

Transverse neocortical slices (300 μm) of the Wistar rat somatosensory cortex were sectioned in ice-cold solution containing (mM): 125 NaCl, 2.5 KCl, 25 glucose, 25 NaHCO_3_, 1.25 NaH_2_PO_4_, 2 CaCl_2_, and 1 MgCl_2_ (carboxygenated with 5% CO_2_/95% O_2_). For paired recordings, slices were transferred to a submerged-type recording chamber containing extracellular solution of the same ionic composition as used for slicing. Individual neurons were identified with an upright microscope (BX-51 WI; Olympus, Japan) at 40× magnification using infrared-differential interference contrast (IR-DIC) microscopy and subsequent fluorescence microscopy.

Signals were recorded using EPC8 amplifiers (HEKA electronics, Lambrecht, Germany), filtered at 3 kHz and digitized at 10 kHz, using an ITC-18 interface (Instrutech, Mineola, NY, USA) and PULSE acquisition software (version 8.21; HEKA electronics, Lambrecht, Germany). Recordings were made at room temperature (20–22°C). Whole-cell current clamp recordings were performed simultaneously from two neurons using borosilicate glass pipettes with resistance of 5–7 MΩ, containing (mM): 105 K gluconate, 30 KCl, 4 Mg-ATP, 10 phosphocreatine, 0.3 GTP, and 10 HEPES (pH 7.3, KOH). The cells with unstable somatic access resistance (>15% changes) or with values larger than 15 MΩ were excluded from the analysis. In synaptically connected neurons, suprathreshold intracellular stimulation of the presynaptic pyramidal cell evoked glutamate-mediated EPSPs in the postsynaptic FS cell. Presynaptic cells were stimulated with a 10 Hz train of 2 or 3 suprathreshold current pulses. Trains were delivered with intervals longer than 7 s. Averages of 50–100 sweeps recorded for each experimental condition were used for analysis. The amplitude of the first EPSP of a train was defined as the difference between the peak of the averaged EPSP and baseline. For the second or third EPSP, the amplitude was the difference between the peak of the averaged EPSP and the baseline measured just before the peak onset. The peak amplitude of the unitary EPSP, evoked by a single presynaptic AP (the first AP in the train) was taken as an index of efficacy, and the percentage of failures as a measure of the reliability of a particular connection. A typical recording of synaptic transmission properties lasted 50 min.

The effect of loading pyramidal neurons with EGTA or BAPTA was measured as the mean amplitude of the first unitary EPSP evoked during a 10 Hz train of presynaptic APs. The individual trains of APs were separated by long intervals (7–10 s). First, 100 responses with buffer-free intracellular control solution were collected to establish a baseline. Subsequently, the presynaptic pipette was retracted and the same pyramidal cell was patched once more with a new pipette, filled with a buffer-containing intracellular solution. After 15–20 min, required for loading the buffer into the terminals, another 100 sweeps were recorded. The change in EPSP amplitude was estimated as the ratio between mean EPSP amplitudes after and before changing the intracellular solution. For changes of intracellular solution during an experiment, a second whole-cell recording of the presynaptic pyramidal neuron was established with a fresh pipette after finishing the first recording (Ohana and Sakmann, 1998; Rozov et al., 2001).

All together this study presents the pooled data from 157 animals and 274 connected cell pairs. In the presynaptic buffer loading and calcium concentration dependance experiments each data point is an average of at least five cells.

All data were analyzed offline using PatchMaster (HEKA, Germany), Igor Pro 6 (WaveMetrics, Portland, OR, USA) and SigmaPlot 13 (Systat Software, San Jose, CA, USA). The statistical significance of differences was assessed with Mann-Whitney rank sum test for two groups with unequal sample sizes, Wilcoxon Signed rank test for pairwise comparison and Kruskal-Wallis one way analysis of variance on ranks for comparing more than two groups. The data are presented as mean ± *SD*, unless otherwise stated.

## Results

### Basic Release Properties in Connections Between L5A Pyramidal Cells and Fast Spiking (FS) Interneurons at P14 and P28 Rats

In the first series of experiments we recorded from neurons of the local microcircuit in layer 5A of the rat somatosensory neocortex formed by presynaptic pyramidal cells and postsynaptic FS interneurons. Previous reports indicate that these synapses undergo a transition from PPD to PPF during the first 4–5 postnatal weeks (Angulo et al., 1999). In order to investigate the underlying mechanisms and to compare pyramidal-to-FS synapses in layer 2/3 and layer 5, we performed paired recordings at these synapses.

Cells were identified based on location and shape of the cell body (observed using infrared microscopy) and their firing pattern. Postsynaptic FS interneurons typically had round somata and showed high-frequency non-accommodating and non-attenuating action potential firing upon depolarizing current injection. The presynaptic neurons were slender tufted (layer 5A) pyramidal cells. These cells were located in upper layer 5 (less than 100 μm from the layer 4) and had a smaller cell body compared to the thick tufted cells (layer 5 pyramidal cells). Upon depolarizing current injection, slender tufted pyramidal cells exhibited regular low-frequency spiking with pronounced accommodation.

**Figure 1:**
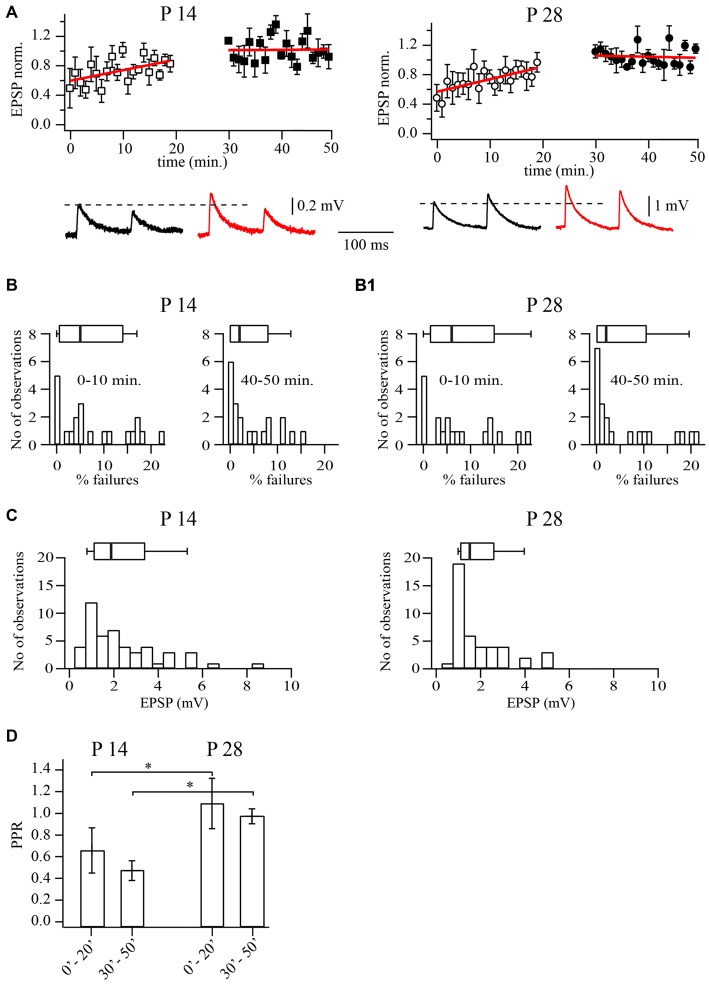
**Prolonged whole-cell recordings change synaptic efficacy at layer 5A pyramidal to fast spiking (FS) cells synapses. (A)** Plots show normalized excitatory postsynaptic potential (EPSP) amplitude distribution during prolonged whole-cell recordings in P14 (left) and P28 (right) animals. Each point is an average of five subsequent sweeps from an individual pair, and the plot is of summarized data from nine pyramidal to FS cell pairs. All EPSP amplitudes were normalized to the mean value from the last 10 min of the experiment (40–50 min). Red lines are the linear fits of the data. Representative averaged traces are shown underneath. **(B)** Failure rate distributions for recordings from pyramidal to FS neuron pairs at P14 **(B)** and P28 **(B1)**. Failure rates were significantly smaller after prolonged dialysis. The histograms show failure rates in the first 10 min of recording, whereas on the right they show failure rates after 40 min of dialysis. **(C)** Distribution of average unitary EPSP amplitudes (30–50 min after whole-cell configuration) recorded in FS neurons following stimulation of the presynaptic pyramidal cell in P14 (left) and P28 (right) animals. **(D)** Bar histogram summarizes the effect of dialysis on PPR at two developmental stages. Box plots above histograms: the box indicates the 25th, 50th (median) and 75th percentiles, the error bars indicate the 10th and 90th percentiles. Asterisks indicate significant difference.

First we evaluated the stability of recordings and synaptic efficacy in the pairs of slender tufted pyramidal to layer 5 FS cell connections at P14 and P28, respectively. We observed a gradual increase of EPSP amplitudes in FS cells during prolonged whole-cell recordings. After the first 20–30 min EPSP amplitudes reached a steady state which was on average almost two times higher than that at the beginning of an experiment. Figure 1A shows a representative trace and summarizes the average time course of the EPSP amplitude for P14 (*n* = 9) and P28 (*n* = 9) animals. Changes in synaptic efficacy might indicate washout of either pre- or postsynaptic factors limiting synaptic transmission. To untangle the pre- or postsynaptic mechanisms underlying the time-dependent change in transmission, we made sequential triple recordings. In the first set of experiments, we continuously recorded from one postsynaptic FS cell while sequentially patching two different presynaptic pyramidal cells. By the time the second pair was obtained, the postsynaptic interneurons had been dialyzed for at least 45 min, ensuring stable internal milieu. However, within the first 15 min of recording with the second pyramidal cell we still observed a gradual increase of EPSPs amplitudes in the postsynaptic FS interneurons (*n* = 5; data not shown). In inverse experiments the presynaptic cell was kept constant while the postsynaptic interneurons were sequentially changed. Here, run-up of responses was observed only in the first pair (*n* = 4; data not shown).

Next we analyzed changes in release probability and paired pulse ratios (PPR; EPSP2/EPSP1). We found that in P14 animals, the averaged PPR values decreased from 0.64 ± 0.2 at the beginning of the experiments to 0.46 ± 0.1 at the end of the experiments (*n* = 20; *p* < 0.01; Wilcoxon signed rank test). A similar reduction of PPRs was observed in P28 animals where values decreased from 1.1 ± 0.2 to 0.94 ± 0.1 (Figure 1D; *n* = 21; *p* < 0.01; Wilcoxon signed rank test). In P28 animals, after prolonged dialysis, facilitation at slender pyramidal to FS cells synapses was washed out almost in all cases. Note that average PPRs in P28 animals were always higher than at P14, both at the beginning or at the end of the experiments (in both cases *p* < 0.01; Mann-Whitney rank sum test).

Analysis of failures revealed highly diverse data between different pairs. As an example, in P14 animals failure rates ranged from 0 to 22%. However, in all pairs with low initial release probability the failure rate decreased significantly after prolonged dialysis of the cell. At P14, the median initial failure rate was 5% and decreased to 2% during the recording (*n* = 20; *p* < 0.01; Wilcoxon signed rank test; Figure 1B). At P28, failure rate medians were 6% (initially) and 2% after 40 min of whole cell dialysis (*n* = 21; *p* < 0.01 Wilcoxon signed rank test; Figure 1B1). Taken together, these data suggest that prolonged dialysis of presynaptic slender tufted cells leads to an increase of release probability. The increase of release probability together with the above-mentioned alterations in synaptic efficacy and PPRs are strongly indicative of a presynaptic origin, most likely the washout of factors controlling calcium levels at the release site.

In the interest of stability, all further experiments were carried out after presynaptic pyramidal cells had been dialyzed for 30 min and EPSP amplitudes in FS interneurons had reached a steady state level. Under these conditions median unitary EPSP amplitudes were 1.89 mV (*n* = 42) and 1.5 mV (*n* = 43; *p* = 0.4; Mann-Whitney rank sum test) in P14 and P28 rats, respectively (Figure 1C).

### Properties of Synaptic Transmission in Connections Between L2/3 Pyramidal Cells and Fast Spiking (FS) Interneurons at P14, P28 and P42 Rats

In these experiments, we recorded from neurons of the local microcircuit in layer 2/3 of rat somatosensory neocortex (Reyes et al., 1998). The presynaptic neurons were pyramidal cells, identified by the shape of the soma and the pattern of frequency accommodation of APs upon depolarizing somatic current injection. The target neurons were non-pyramidal, multipolar interneurons, as viewed with infrared video-microscopy and characterized by non-accommodating FS firing patterns (Reyes et al., 1998; Rozov et al., 2001).

**Figure 2:**
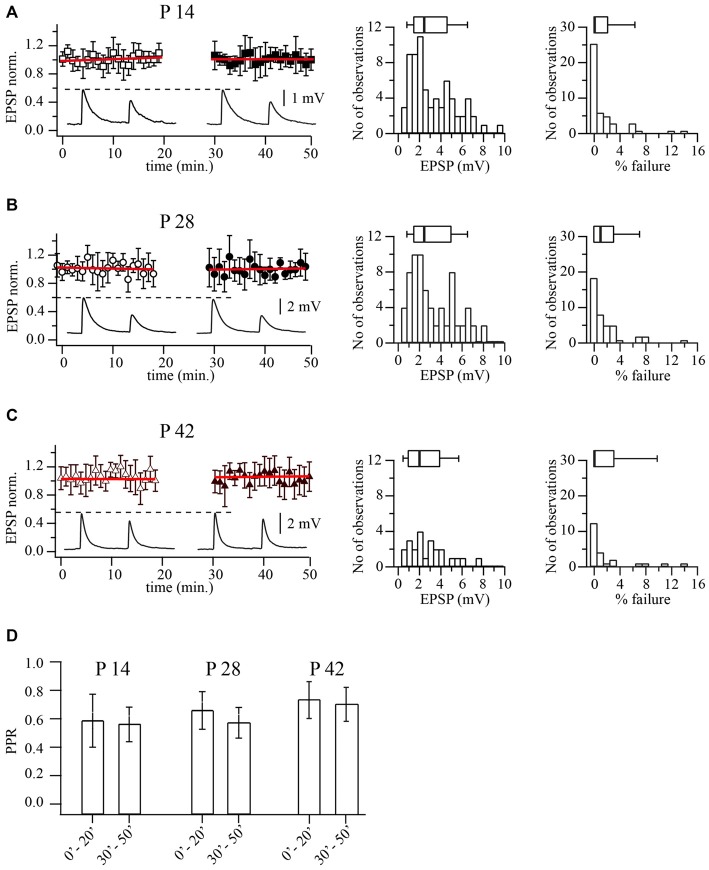
**Prolonged whole-cell recordings do not change synaptic efficacy at layer 2/3 pyramidal to FS cell synapses. (A)** Plot shows normalized EPSP amplitude distribution during prolonged whole-cell recordings in P14 animals. Each point is an average of five subsequent sweeps from an individual pair, and the plot summarized data from nine pyramidal to FS cell pairs. All EPSP amplitudes were normalized to the mean value from the last 10 min of the experiment (40–50 min). Red lines are the linear fits of the data. Representative averaged traces are shown underneath. Histograms display distribution of average unitary EPSP amplitudes (middle) and failure rate distribution (right) recorded in FS neurons following stimulation of the presynaptic pyramidal cell in P14 animals. Box plots above histograms: the box indicates the 25th, 50th (median) and 75th percentiles, the error bars indicate the 10th and 90th percentiles. **(B)** The same as in **(A)** obtained for P28 animals. Scatter plot summarizes data from 10 cell pairs. **(C)** The same as in **(A)** obtained for P42 animals. Scatter plot summarizes data from seven cell pairs. **(D)** Bar histogram summarizes the effect of dialysis on PPR at three developmental stages.

Since cortical lamination is formed in an inside-out fashion, development of synaptic properties at connections formed by layer 2/3 pyramidal cells might be delayed relative to those in layer 5. To test this notion we compared release probability, synaptic efficacy and PPRs at layer 2/3 pyramidal to FS cell synapses in P14, P28 and P42 animals. In contrast to the results described above for layer 5 pairs, in most experiments, unitary EPSP amplitudes and PPRs remained constant during the recording time (Figure 2). Although amplitude distributions for all ages were broad, median amplitudes were very similar, being 2.43 mV (*n* = 69), 2.43 mV (*n* = 71) and 1.98 mV (*n* = 27) for P14, P28 and P42 respectively (*p* = 0.42; Kruskal-Wallis one way analysis of variance on ranks; Figures 2A–C).

Next we analyzed reliability of release by measuring the percentage of failures of the presynaptic AP to evoke an EPSP. Figures 2A–C shows the distribution of failures in pyramidal to FS neurons connections at three developmental stages. Again no significant changes in failure rates were found. Median values were; 0% at p14 (*n* = 69), 1% at P28 (*n* = 71) and 0% at P42 (*n* = 27; *p* = 0.63; Kruskal-Wallis one way analysis of variance on ranks). In all three ages, APs rarely failed to elicit an EPSP in multipolar neurons, indicating a very reliable synaptic connection.

Previous evidence has indicated marked pronounced short-term depression at synapses from layer 2/3 pyramidal to FS cells (Reyes et al., 1998; Rozov et al., 2001). In line with this observation, the average PPR was 0.58 ± 0.12 (*n* = 69) for P14, 0.64 ± 0.11 (*n* = 71) for P28 and 0.71 ± 12 (*n* = 27) for P42 rats (*p* = 0.31; Kruskal-Wallis one way analysis of variance on ranks). To rule out the possibility that gradual deterioration of the cells was responsible for the synaptic depression, we analyzed PPRs at the beginning (first 10 min.) and the end (last 10 min.) of each experiment. In most of the experiments PPRs remained constant (Figure 2D). Thus in layer 2/3, the basic properties of synaptic release and type of short term plasticity at pyramidal to FS cells connections do not change between the second and sixth postnatal weeks. Therefore all subsequent experiments were carried out on P14 and P28 animals.

### Calcium Concentration Dependence of Efficacy at Layer 2/3 Pyramidal to FS Cells

It has been shown that differential dependance of release on extracellular calcium concentration [Ca^2+^]_o_ could indicate structural differences in release zone organization in facilitating and depressing terminals. It has also been demonstrated that different degrees of saturation of the presynaptic calcium sensor occur during single APs (Rozov et al., 2001). Therefore we tested whether the observed developmental change in PPR goes along with changes in the relation between release probability and [Ca^2+^]_o_. Extracellular calcium concentration was varied between 1, 2, 3 and 4 mM, respectively. In order to compare across different cell pairs, averaged EPSP amplitudes and PPRs at each [Ca^2+^]_o_ were normalized to the values obtained with the control calcium concentration (2 mM).

**Figure 3:**
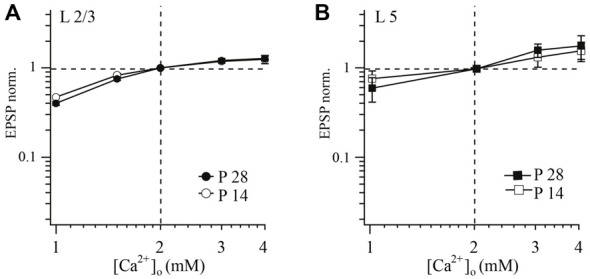
**Effect of [Ca^2+^]_o_ on synaptic efficacy at layer 2/3 and layer 5A pyramidal cell to FS cell synapses.** The relative change of unitary EPSP amplitude following changes of [Ca^2+^]_o_ in connections of layer 2/3 **(A)** or layer 5 **(B)** pyramidal cells with FS cells at two different ages (P14 and P28) plotted on a double logarithmic scale. In each experiment [Ca^2+^]_o_ was either increased or decreased from control (2 mM). [Mg^2+^]_o_ was kept constant at 1 mM. EPSPs measured at different [Ca^2+^]_o_ were normalized to the values obtained at 2 mM [Ca^2+^]_o_ from the same experiment.

As illustrated in Figure 3A, dependances of release probability from layer 2/3 pyramidal-to-FS cells terminals on [Ca^2+^]_o_ in P14 and P28 animals were very similar. EPSP amplitude was already very close to saturation at the normal [Ca^2+^]_o_ of 2 mM. The averaged data points could be fitted satisfactorily by a Hill equation with an exponent of four and half-effective concentrations (*K*_1/2_) of [Ca^2+^]_o_ amounting to 1.09 and 1.1 mM, for P14 and P28 respectively.

In both P14 and P28 rats, reduction of [Ca^2+^]_o_ led to an increase of PPR, and synaptic depression was most pronounced at higher [Ca^2+^]_o_ (data not shown). However, no statistical difference in these parameters was observed between younger and older animals (at all tested [Ca^2+^]_o_ concentrations *p* > 0.05; Mann-Whitney rank sum test).

### Calcium Concentration Dependence of Efficacy at Layer 5A Pyramidal to FS Cell Synapses

Next we examine whether a similar dependance between [Ca^2+^]_o_ and release probability is present at layer 5 pyramidal to FS neurons. Using the same experimental protocol as in layer 2/3 we found no significant differences in calcium-dependance of release between young (P14) and mature (P28) animals at slender pyramidal-to-FS cell terminals (at all tested [Ca^2+^]_o_ concentrations *p* > 0.05; Mann-Whitney rank sum test; Figure 3B). Averaged data points were fitted using a Hill equation, yielding an exponent of four and half-effective concentrations *(K_1/2_)* of 2.44 and 2.29 mM [Ca^2+^]_o_ for P14 and P28, respectively.

Similar to the layer 2/3 connection, reduction of [Ca^2+^]_o_ led to an increase in PPR and depression was most pronounced at high [Ca^2+^]_o_ in both P14 and P28 rats. However, we did not find any statistical difference between slices from younger and older animals. Since sensitivity of release to [Ca^2+^]_o_ at layer 5 pyramidal to FS cell remains the same during the second two postnatal weeks, facilitation at P28 cannot be explained by the change of the calcium sensor sensitivity.

### Differential Effects of EGTA and BAPTA on Release at Layer 2/3 Pyramidal Cell to FS Cell Synapses

Sensitivity of transmission to exogenously loaded buffers can be used to probe the presynaptic determinants of release probability and short-term plasticity. For example, synapses with a long diffusional distance between calcium channels and calcium sensors are sensitive to both fast and slow exogenous buffers. On the other hand, if calcium channels are located closely to calcium sensors, the effect of calcium buffers is much less pronounced (Neher, 1998). Buffer sensitivity has been successfully used to investigate differences between facilitating and depressing synapses (Rozov et al., 2001). Here, we used the same approach to check whether the spatial arrangement between the calcium source and calcium sensor in layer 2/3 pyramidal cell terminals onto FS cells undergoes developmental modifications (for details, see “Materials and Methods” Section).

**Figure 4:**
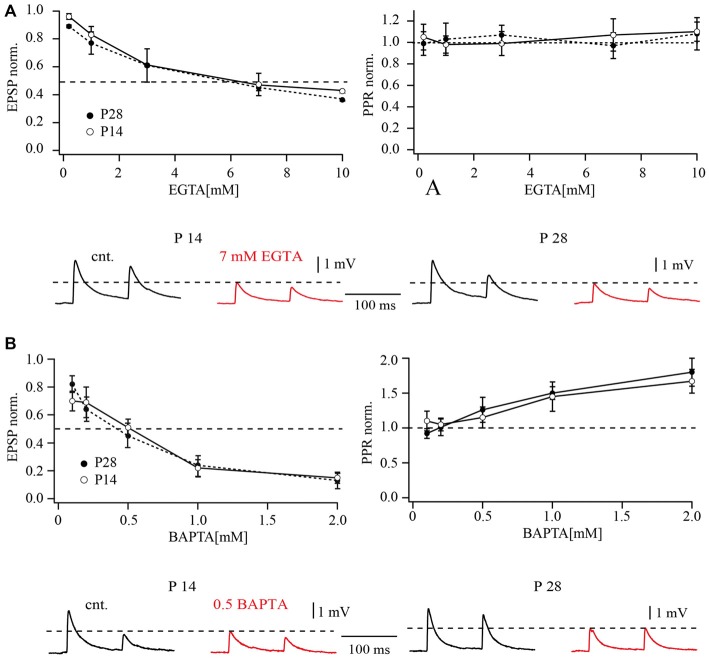
**Effects of buffer loading on release from layer 2/3 pyramidal cell terminals on FS cells. (A)** The left plot shows the concentration-dependent effect of presynaptic EGTA loading on the mean amplitude of unitary EPSP at layer 2/3 pyramidal to FS cell synapses. Mean EPSP amplitude was normalized to control values determined in the same connection before loading with buffer. The half-effective concentrations (when the mean EPSP amplitude was reduced to 50% of the control; dashed lines) were around 7 mM EGTA in P14 and P28 rats. The plot on the right shows the concentration-dependent effect of presynaptic EGTA on depression of EPSPs recorded in FS cells. The EPSP2/EPSP1 ratio (normalized to control value) is plotted as a function of buffer concentration in the pipette recording from the presynaptic pyramidal cell. The traces underneath demonstrate the effects of presynaptic EGTA loading on unitary EPSPs recorded from FS cells at P14 (left) and P28 (right). Control averaged EPSPs are black and EPSPs recorded after loading of the pyramidal neuron with 7 mM EGTA are red. **(B)** The left plot shows the concentration-dependent effect of presynaptic BAPTA loading on the mean amplitude of unitary EPSP at pyramidal to FS cell synapses. The half-effective concentrations were 0.5 mM BAPTA in P14 and P28 rats. The plot on the right shows the concentration-dependent effect of presynaptic BAPTA on depression of EPSPs recorded in FS cells. Traces underneath demonstrate effects of presynaptic 0.5 mM BAPTA loading on unitary EPSPs recorded from FS cells at P14 (left) and P28 (right). Control averaged EPSPs are black and EPSPs recorded after buffer loading are red.

Release from pyramidal cell terminals to FS cells was suppressed by EGTA in a concentration dependent manner. This reduction was already significant at a concentration of 0.2 mM. Figure 4A compares the dependance of release on EGTA concentration for P28 layer 2/3 pyramidal to FS cell with data published for the same type of synapses in P14 animals (Rozov et al., 2001). Mean EPSP amplitudes were normalized to the control averaged EPSP amplitude before buffer loading and plotted as a function of buffer concentration. The half effective concentration of EGTA for both P14 and P28 animals was very similar, with values around 7 mM. At both ages, EGTA did not have any statistically significant effects on PPD (*p* > 0.05; Wilcoxon signed rank test, at all tested EGTA concentrations). PPRs obtained upon buffer loading were normalized to control PPR values, to minimize the effect of scatter in the initial synaptic depression level between different cell pairs (Figure 4A, right plot).

Loading of the fast exogenous buffer BAPTA had a much stronger effect on synaptic release at pyramidal to FS cells compared to EGTA. At P28, the half-effective concentration of BAPTA was about 0.5 mM, comparable to that measured previously in P14 animals (Rozov et al., 2001). Dose-response curves for the effect of BAPTA on synaptic release are shown in Figure 4B. At both ages BAPTA could efficiently suppress synaptic depression at concentrations higher than 0.5 mM (Figure 4B, right plot). Together, these data largely exclude major spatial rearrangements of calcium sources and calcium sensors at the vesicle release site.

### Differential Effects of EGTA and BAPTA on Release at Layer 5A Pyramidal Cells with FS Cell Synapses

**Figure 5:**
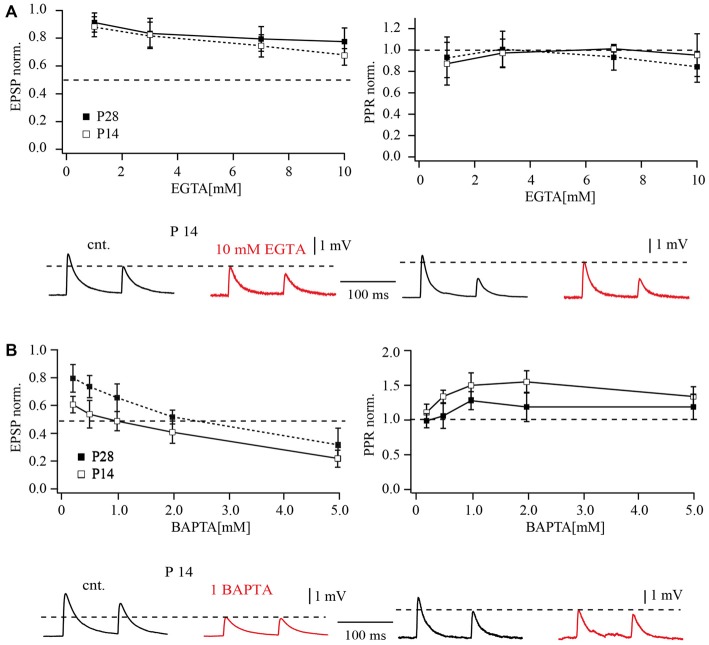
**Effects of buffer loading on release from layer 5A pyramidal cell terminals to FS cells. (A)** The concentration-dependent effect of presynaptic EGTA loading on the mean amplitude of unitary EPSP evoked by AP stimulation of terminals contacting FS cells in P14 and P28 rats (left). Mean EPSP amplitude was normalized to control values determined in the same connection before loading with buffer. The plot on the right shows the concentration-dependent effect of presynaptic EGTA on depression of EPSPs recorded in FS cells. The EPSP2/EPSP1 ratio (normalized to control value) is plotted as a function of buffer concentration in the pipette recording from the presynaptic pyramidal cell. Traces underneath demonstrate effects of presynaptic EGTA loading on unitary EPSPs recorded from FS cells at P14 (left) and P28 (right). Control averaged EPSPs are black and EPSPs recorded after loading of pyramidal neuron with 10 mM EGTA are red. **(B)** The left plot shows the concentration-dependent effect of presynaptic BAPTA loading on the mean amplitude of unitary EPSP at layer 5 pyramidal to FS cell synapses. The half-effective concentrations were 1 mM BAPTA and 2 mM BAPTA in P14 and P28 rats respectively. The plot on the right shows the concentration-dependent effect of presynaptic BAPTA on depression of EPSPs recorded in FS cells. Traces underneath demonstrate effects of presynaptic 1 mM BAPTA loading on unitary EPSPs recorded from FS cells at P14 (left) and P28 (right). Control averaged EPSPs are black and EPSPs recorded after buffer loading are red.

For comparison, we tested the effect of calcium buffers on synaptic transmission between slender tufted pyramidal cells and FS neurons in layer 5. Figure 5A shows the effect of the slow calcium buffer EGTA on synaptic release at both ages, P14 and P28. EGTA had relatively small effects on synaptic release. When 10 mM EGTA was loaded into the presynaptic cell, unitary EPSP amplitudes were reduced to 62 ± 11% of the control value in P14. In P28 animals the effect of 10 mM EGTA appeared to be even less pronounced (78 ± 14% of control; Figure 5A). The apparent decrease of EGTA-sensitivity with increasing age was, however, not significant (*p* > 0.05; Mann-Whitney rank sum test).

Loading of the fast exogenous buffer BAPTA had a much stronger effect on synaptic release at layer 5A pyramidal to FS cell connections than EGTA. A comparison of dose response curves for P14 and P28 animals is shown in Figure 5B. In contrast to EGTA, the effects of BAPTA were significantly less pronounced at later developmental stages. The half effective concentration for BAPTA in P14 animals was around 1 mM which suppressed EPSPs to 0.49 ± 0.12% of control (Figure 5B). In older animals, 2 mM BAPTA was required to suppress release by half (52 ± 10% of control EPSP amplitude). Thus, during maturation of slender tufted pyramidal to FS cell terminals, sensitivity of synaptic release to exogenously loaded buffers is reduced. This finding indicates a shortening of the diffusional distance between calcium channels and calcium sensors. However, this assumption is in apparent contradiction with the reduction of release probability and increased PPRs in P28. One possible explanation might be that reduction in diffusional distance is accompanied by a change in the type of calcium channels.

### Calcium Channel Subtypes Mediating Release at Layer 2/3 Pyramidal to FS Cell Synapses

Presynaptic calcium channel subtypes can change during development, leading to significant changes in the efficacy and dynamics of synaptic transmission. Specifically, the expression of N-type calcium channels keeps growing during the first postnatal weeks and reaches maximum levels at ~P28 in the rat hippocampus and cortex (Jones et al., 1997). In the rat striatum, N-type calcium channel expression is significantly down regulated during the third postnatal week (Martella et al., 2008). Therefore, we compared the contribution of different subtypes of calcium channels to synaptic release at layer 2/3 pyramidal to FS cell synapses in P14 and P28 rats using specific calcium channel blockers.

**Figure 6:**
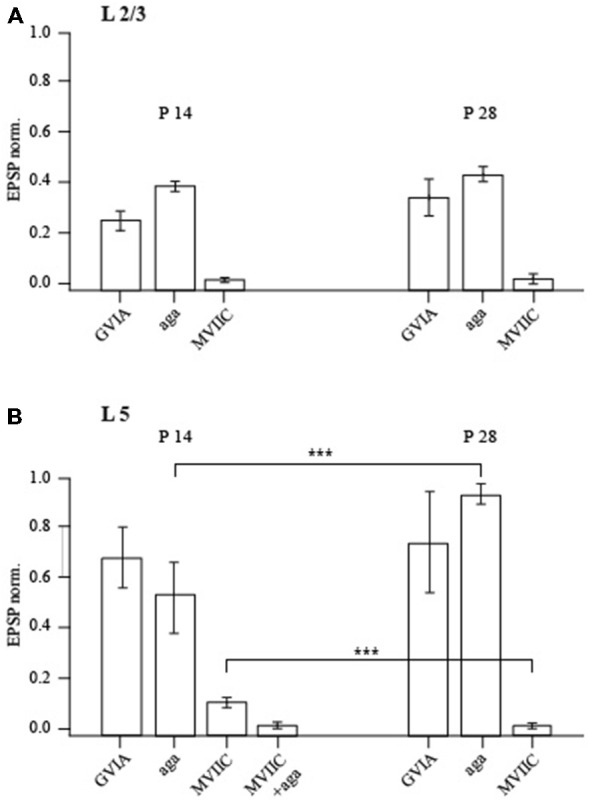
**Effect of calcium channel-blocking toxins on synaptic efficacy.** Bar histograms show the effects of P/Q-type, N-type and P/Q/N-type calcium channel-specific toxins on evoked unitary EPSPs recorded in layer 2/3 **(A)** and layer 5 **(B)** FS neurons in P14 and P28 rats. The ordinate represents (in %) the ratio of mean EPSP amplitude in the steady state during and before toxin application. Asterisks indicate significant difference.

Bath application of 100 nM of the P/Q-type calcium channel blocker agatoxin IVA (Aga) reduced the amplitude of unitary EPSPs relative to control values to 38 ± 3% (*n* = 7; *p* < 0.01; Wilcoxon signed rank test) and to 41 ± 4% (*n* = 6; *p* < 0.01; Wilcoxon signed rank test), at P14 and P28 respectively (Figure 6). Of note, this toxin has much higher affinity to P-type channels with a *K*_D_ of about 1 nM than to Q-type channels with a *K*_D_ of 100 nM. Application of the P/Q- and N-type channel blocker ω-conotoxin (MVIIC) almost entirely blocked synaptic transmission. In the presence of 1 μM of MVIIC, unitary EPSP amplitudes were reduced to 2.1 ± 0.4 (P14; *n* = 6; *p* < 0.01; Wilcoxon signed rank test) and to 2.3 ± 1.5% (P28; *n* = 6; *p* < 0.01; Wilcoxon signed rank test) of the control value. This toxin blocks Q-type channels more efficiently (*K*_D_ of about 5 nM) than P-type channels (*K*_D_ of about 500 nM), suggesting a significant contribution of Q-type channels to release (Zhang et al., 1993). The EPSP that remained after adding saturating toxin concentrations could be blocked by 50 μM Cd^2+^ (at both ages *p* < 0.01; Wilcoxon signed rank test). The remaining release is most probably due to Ca^2+^ influx via R-type calcium channels, since it was completely blocked by 100 μM Ni^2+^ (at both ages *p* < 0.01; Wilcoxon signed rank test). Application of the specific N-type channel blocker ω-conotoxin (GVIA) at 1 μM suppressed release to 25 ± 4% at P14 (*n* = 6; *p* < 0.01; Wilcoxon signed rank test) and to 32 ± 7% at P28 (*n* = 5; *p* < 0.01; Wilcoxon signed rank test) of control values, again indicating that N-type channels strongly contribute to evoked release in pyramidal to FS cell connections at both ages (Figure 6A). Thus, at P14 and at P28 chemical transmission between pyramidal and FS neurons is mostly mediated by P/N/Q-type calcium channels. Taken together our data shows that in layer 2/3 pyramidal to FS cell synapses a number of important parameters stay constant between P14 and P28. These include basic release probability, [Ca^2+^]_o_ dependance, sensitivity to exogenously loaded buffers and the relative contribution of presynaptic P/N/Q-type calcium channels.

### Calcium Channel Subtypes Mediating Release at Layer 5A Pyramidal to FS Cell Synapses

In P14 animals, EPSPs in FS cells evoked by stimulation of layer 5A pyramidal cells were blocked to 52 ± 12% (*n* = 5; *p* < 0.01; Wilcoxon signed rank test) of the control value by bath application of 100 nM Agatoxin, a P/Q-type calcium channel blocker. Surprisingly 1 μM of GVIA, a specific N-type channel blocker, had only mild effects on release (62 ± 10.2% of control value; *n* = 7; *p* < 0.05; Wilcoxon signed rank test) indicating a limited contribution of N-type channels to evoked release at this connection. Moreover, in the presence of the P/Q- and N-type channel blocker MVIIC (1 μM) 11 ± 2% (*n* = 6; *p* < 0.001; Wilcoxon signed rank test) of the initial EPSP amplitude remained. The remaining release is most probably due to P-type calcium channels, since a mixture of MVIIC (1 μM) and Agatoxin (100 nM) almost entirely blocked synaptic transmission to 2 ± 1% of control value (*n* = 5; *p* < 0.001; Wilcoxon signed rank test; Figure 6B). Thus, in P14 animals synaptic transmission between slender tufted pyramidal and FS neurons is mediated predominately by P/Q-calcium channels, with a small contribution of N type channels.

In P28 animals the effect of GVIA on synaptic release was small (73 ± 18% of control value; *n* = 7; *p* < 0.05; Wilcoxon signed rank test) and similar to that in young animals. In contrast to P14, application of Agatoxin did not have any significant effect on synaptic release at P28 (92 ± 4% of control EPSP remained; *n* = 8; *p* > 0.05; Wilcoxon signed rank test). This indicates a strongly reduced contribution of P-type calcium channels to synaptic release at layer 5A pyramidal to FS cell synapses. On the other hand, application of MVIIC almost entirely blocked EPSPs down to 1.2 ± 1% (*n* = 6; *p* < 0.001; Wilcoxon signed rank test) of the control value, suggesting an increased contribution of Q type calcium channels (Figure 6). In both P14 and P28 animals, the residual release remaining after application of MVIIC and Agatoxin or of MCVII alone was mediated by R type calcium channels, since it could be completely blocked by 50 μM Cd^2+^ (*n* = 5; *p* < 0.01; Wilcoxon signed rank test). Thus, based on the differential effects of Agatoxin and MVIIC on evoked EPSP amplitudes and taking into account the differences in *K*_D_, we conclude that at P28 in layer 5A pyramidal to FS cells terminals, release is triggered almost solely by calcium entry through Q type calcium channels.

## Discussion

### No Developmental Changes at Layer 2/3 Pyramidal to FS Cell Connections

We investigated local layer 2/3 circuits composed of presynaptic pyramidal cells and postsynaptic parvalbumin positive FS interneurons (Reyes et al., 1998; Rozov et al., 2001). It has been reported that at P28 in connections between pyramidal cells in layer 2/3, as well as in connections between layer 2/3 pyramidal cells and layer 5 pyramidal cells, slight facilitation substitutes the pronounced depression observed in P14 rats (Reyes and Sakmann, 1999). Therefore, one might have expected similar changes in other circuits where layer 2/3 pyramidal cells act as the presynaptic partner. However, our analysis of layer 2/3 pyramidal to FS cell connections at two different ages (P14 and P28) did not reveal any statistical differences in a number of parameters including: initial release probability, unitary EPSP amplitude, PPRs, presynaptic calcium channel composition, and sensitivity to [Ca^2+^]_o_ and exogenously loaded buffers. These results suggest that developmental modulations of local microcircuits are connection-specific.

### A Change in Ca^2+^ Channel Subtypes Underlies Developmental Modulation of Release Properties at Layer 5A Pyramidal to FS Cells Connections

Angulo et al. (1999) reported a developmental switch from short-term depression to short-term facilitation in the layer 5 pyramidal to FS cell connections. Although, the presynaptic neurons described in that study belonged to a different subtype (thick tufted pyramidal cells), here we found similar changes take place in another layer 5 circuit formed by slender tufted pyramidal cells and the same type of interneurons. Note, that regardless of the origin of the excitatory input, FS interneurons receive a similar pattern of excitation: more depressing in young than old animals. Interestingly, the time point of the developmental switch in the mode of short-term synaptic plasticity is also similar in both types of connections. One possible explanation for the coincidence of the developmental alterations in two distinct types of terminals targeting the interneuronal subclass might be a common retrograde signal coming from the postsynaptic cell to the two classes of presynaptic cells. In this study, we made an attempt to find out a structural basis for the developmental changes in layer 5 pyramidal cells in the terminals. The major difference between P14 and P28 animals was distinct sensitivity to calcium channel blockers. In this study, we took advantage of the differences in the *K*_D_ of P- and Q-type calcium channels to Agatoxin and ω-conotoxin MVIIC. Q-type channels are 100 fold more sensitive to MVIIC (*K*_D_ = 5 nM) than P-type (*K*_D_ = 500 nM). P-type calcium channels are blocked at much lower concentrations of Agatoxin (*K*_D_ = 1 nM) than Q-type (*K*_D_ = 100 nM). Thus, at the concentration used in our experiments MVIIC mainly blocked Q-type channels, while Agatoxin acted on the P-type channels. Our results showed that in younger animals, release was mediated by both P- and Q- type channels. A significant contribution of P-type channels is indicated by the fact that only a mixture of MVIIC and Agatoxin resulted in an almost complete blockade of release. In P28 animals Agatoxin did not have any effect on synaptic transmission, suggesting that the role of the P-type calcium channels in transmitter release is negligible.

Surprisingly we found that the developmental increase in PPRs in layer 5A pyramidal to FS cell synapses is accompanied by reduced sensitivity to calcium chelators. The latter would imply a more compact organization of the release sites, with calcium channels more tightly coupled to the calcium sensor. However, if calcium entry remained the same this should lead to an increased release probability, and consequently increased synaptic depression. The most rational way to explain this contradiction is to assume that channels are indeed closer to the sensor, but they provide less calcium at the release site. For example, they might be fewer in numbers.

Another remarkable feature of layer 5A pyramidal cells is the existence of presynaptic washable factors that can reduce synaptic release, since prolonged dialysis of presynaptic slender tufted cells leads to increased release probability and a reduction in PPRs. One possible explanation might be washout of mobile endogenous calcium buffers from presynaptic terminals (Blatow et al., 2003). Indeed, recently it has been shown that layer 5A pyramidal cells express calretinin with the peak of immunoreactivity at P8. However, calretinin expression in the pyramidal neurons decreases at P14 and disappears by P30 (Liu et al., 2014). Therefore calretinin washout can only partly underlay the increase in EPSPs.

### Differences in Release Properties Between Layer 5A and Layer 2/3 Pyramidal to FS Cell Connections

Although both of the microcircuits studied here are formed by similar neuronal cell types: presynaptic pyramidal cells and postsynaptic FS interneurons, they differ in a number of their presynaptic release properties. Layer 2/3 pyramidal cell terminals are more sensitive to any manipulation of the intraterminal calcium concentration. Lowering of extracellular calcium leads to stronger reduction of EPSP amplitudes at layer 2/3 pyramidal to FS cells synapses (EPSP norm. 0.31, 1 mM [Ca^2+^]_o_, P28) than at those in layer 5 (EPSP norm. 0.63, 1 mM [Ca^2+^]_o_, P28). The layer 2/3 pyramidal cell terminals also show much higher sensitivity to exogenously loaded buffers compared to layer 5 pyramidal cells. These observations are in apparent contradiction with the fact that layer 2/3 pyramidal cell inputs to FS cells are characterized by significantly higher unitary EPSP amplitudes (medians 2.43 vs. 1.5; L2 vs. L5; P28; *p* = 0.007; Kruskal-Wallis one way analysis of variance on ranks) and a slightly lower failure rate (1% vs. 0%; L2 vs. L5; P28) compared to layer 5 pyramidal to FS cell connections. A possible explanation for this might be the different calcium micro-domain organization in layer 2/3 and 5 pyramidal cell terminals. We can speculate that on the one hand the distance for diffusion between calcium channels and the calcium sensor is longer in layer 2/3 terminals than in layer 5 terminals. This would explain the higher sensitivity of release at layer 2/3 synapses to changes in intracellular calcium dynamics. On the other hand, a higher release probability implies higher calcium concentration at the release site, which might be due to a higher density of calcium channels or lower endogenous buffer capacity in layer 2/3 pyramidal cell terminals compared to terminals of layer 5A pyramids. The first assumption is indirectly supported by our finding that terminals of layer 2/3 and layer 5 pyramids express different subtypes of calcium channels, that may lead to a distinct spatial arrangement of calcium sensor and calcium channels at the release sites. A gradual increase in EPSP amplitudes at layer 5 synapses during prolonged recordings is indicative of washout of compounds, possibly calcium chelating molecules that affect calcium signaling.

Finally, the two synapses studied differ in their developmental profiles. While layer 2/3 pyramidal to FS cell terminals do not show any significant developmental changes, excitatory inputs from layer 5A pyramids to FS interneurons undergo drastic modification, changing them from strongly depressing to slightly facilitating synapses. All together our data shows that developmental mechanisms are different in these different synapses and therefore, detailed studies of the maturation of a given type of connection are critical for our understanding of neuronal network functional connectivity.

### Possible Functional Implications of Layer Specific Properties at Pyramidal to FS Cells Synapses

FS basket interneurons have been shown to play a key role in the generation of gamma rhythms in the neocortex and hippocampus (Fuchs et al., 2007; Cardin et al., 2009; Sohal et al., 2009). Two models are proposed as possible mechanisms for generating gamma oscillations. According to the first, a network consisting of only mutually connected inhibitory interneurons with distinct intrinsic firing properties and GABA_A_ receptor kinetics, can upon sufficient excitatory drive generate sustained oscillations. The second model is based on reciprocal connections between pools of excitatory pyramidal and inhibitory neurons. In such a network fast excitation and delayed feedback inhibition alternate leading to persistent rhythmic activity (for review, see Buzsaki and Wang, 2012). Indeed, the present findings suggest that excitatory drive of FS cells by local excitatory neurons is an important requirement for the generation of gamma oscillations (Middleton et al., 2008; Tiesinga and Sejnowski, 2009). Furthermore, in animal models of human psychiatric illness, weakening of excitatory input to FS cells, leads to impaired gamma oscillations (Cunningham et al., 2006; Sauer et al., 2015).

Our data suggest that local excitatory synapses between pyramidal cell and FS interneuron in layer 2/3 have significantly higher unitary EPSP amplitudes compared to layer 5. In addition, layer 2/3 connections are characterized by strong synaptic depression. This implies that during rhythmic or bursting activity the probability of recruiting downstream FS neurons is highest at the beginning of the burst. A similar scenario of crosstalk between pyramidal and FS cell is expected in young animals due pronounced PPD. However, in older rat when these synapses switch the mode of short term plasticity to facilitation, shifting efficiency of synaptic transmission towards latter AP in the burst. All together this might result in higher synchrony in upper layer 2/3 compared with the deep layers of the cortex. Indeed, it has been shown with *in vivo* experiments carried on rhesus monkeys that gamma in superficial layers is characterized by significantly higher synchrony compared to deep layers (Buffalo et al., 2011). It was also found that spike-field coherence in the gamma frequency range was largely confined to the superficial layers, while the deep layers showed maximal coherence at low frequencies. The layer-specific differences in efficacy and short term synaptic plasticity at local pyramidal to FS interneuron synapses might contribute to laminar specificity of network activities.

## Author Contributions

OV, acquisition of the data and analysis and interpretation of the data. FV, acquisition of the data and analysis and interpretation of the data. YZ, acquisition of the data and analysis of the data. MM, analysis of the data. AD, study conception and design. Drafting of manuscript. AR, study conception and design. Acquisition of the data and analysis and interpretation of the data and drafting of manuscript.

## Conflict of Interest Statement

The authors declare that the research was conducted in the absence of any commercial or financial relationships that could be construed as a potential conflict of interest.

The reviewer Vincenzo De Paola and handling Editor Thomas Knöpfel declared their shared affiliation, and the handling Editor states that the process nevertheless met the standards of a fair and objective review.
